# Self-management in early-stage dementia: a pilot randomised controlled trial of the efficacy and cost-effectiveness of a self-management group intervention (the SMART study)

**DOI:** 10.1186/1745-6215-15-74

**Published:** 2014-03-08

**Authors:** Catherine Quinn, Daniel Anderson, Gill Toms, Rhiannon Whitaker, Rhiannon Tudor Edwards, Carys Jones, Linda Clare

**Affiliations:** 1 School of Psychology, Bangor University, Bangor, Gwynedd LL57 2AS, UK; 2 The Retreat, Heslington Road, York YO10 5BN, UK; 3 North Wales Organisation for Randomised Trials in Health, Y Wern, Bangor University, The Normal Site, Holyhead Road, Gwynedd LL57 2PZ, UK; 4 Centre for Health Economics and Medicines Evaluation, Bangor University, Dean Street, Gwynedd LL57 1UT, UK

**Keywords:** Alzheimer’s disease, Vascular dementia, Caregiver, Self-efficacy, Well-being, Support

## Abstract

**Background:**

The possibility of living well with a long-term condition has been identified as centrally relevant to the needs of people living with dementia. Growing numbers of people with early-stage dementia are contributing accounts that emphasise the benefits of actively engaging in managing the condition. Self-management interventions share the common objectives of educating about the condition, optimising well-being, enhancing control over the situation and enabling people to take more responsibility for managing the condition. Benefits of such an approach can include improved knowledge, self-efficacy, health status, and better performance of self-management behaviours. However, there is only preliminary evidence that people with early-stage dementia can benefit from such interventions.

**Methods:**

This feasibility study involves the development of a self-management group intervention for people with early-stage Alzheimer’s disease, vascular dementia or mixed Alzheimer’s and vascular dementia. This study is a single-site pilot randomised-controlled trial. Forty-two people with early stage dementia, each with a caregiver (family member/friend), will be randomised to either the self-management group intervention or to treatment as usual.

The self-management group intervention will involve eight weekly sessions, each lasting 90 minutes, held at a memory clinic in North Wales. All participants will be re-assessed three and six months post-randomisation. This study is intended to supply an early evaluation of the self-management intervention so that a full scale trial may be powered from the best available evidence. It will assess the feasibility of the intervention, the study design and the recruitment strategies. It will estimate the parameters and confidence intervals for the research questions of interest. The primary outcome of interest is the self-efficacy score of the person with dementia at three months post-randomisation. Secondary outcomes for the person with dementia are self-efficacy at six months post-randomisation and cognitive ability, mood and well-being at three and six months post-randomisation. Secondary outcomes for caregivers are their distress and stress at three and six months post-randomisation. The cost-effectiveness of the intervention will also be examined.

**Discussion:**

This study will provide preliminary information about the feasibility, efficacy and cost-effectiveness of a self-management group intervention for people in the early stages of dementia.

**Trial registration:**

Current Controlled Trials, ISRCTN02023181.

## Background

Enabling self-management for people with long-term health conditions is a continuing policy aim [1,2]. Self-management has been defined as an ‘individual’s ability to manage the symptoms, treatment, physical and psychosocial consequences and life style changes inherent in living with a chronic condition’ [3]. The possibility of living well with a long-term condition has been identified as centrally relevant to the needs of people with dementia and is a key tenet of current policy [4]. In the absence of a cure for dementia, there is a role for psychosocial interventions in promoting optimal functioning [5].

Given the cognitive and functional decline involved in dementia, self-management is most relevant in the early stages, where the emphasis is on managing and living well with the condition [6-8]. Offering suitable interventions to people with early-stage dementia could delay admission to residential care and add to the cost-effectiveness of services; in other long-term health conditions, participation in self-management programmes leads to a universal reduction in service costs that remains evident over time [9].

The findings from a review of self-management interventions in other long-term conditions have suggested that self-management approaches provide benefits for participants in terms of improved knowledge, performance of self-management behaviours, self-efficacy and aspects of health status [3]. A recent review of various self-management and educational interventions across a range of conditions concluded that assisting people to become more knowledgeable about and to develop basic skills in managing their health condition could result in physical and psychological benefits [10]. In terms of health conditions, the paper concluded that self-management had definite benefits for people suffering from asthma and that this approach showed promise in areas such as diabetes, epilepsy and mental health.

While there is evidence for the benefits of self-management interventions, the theoretical bases of these interventions are often not explained [11]. The most common conceptualisation of self-management [12] is based on social cognitive theory [13,14]. Bandura proposed that behaviour is influenced by goals, level of self-efficacy, outcome expectations and various sociocultural factors. Most empirical work has focused on the self-efficacy component of this model. Self-efficacy is defined as the person’s belief that s/he can perform a specific action in a particular situation. Self-regulation offers another theoretical basis for self-management. This theory proposes that self-observation, self-evaluation and self-reactions are the processes by which people learn to deal with complex environments and develop problem-solving strategies [15].

In relation to dementia, there is evidence to suggest that people with early-stage dementia are usually able to identify some issues that they would like to manage better [16]. This offers an avenue for a sensitive and tailored approach to encourage individuals with early-stage dementia to draw on their resources and on support from others to make positive changes. Approaches such as support groups [17], psychotherapy groups [18], goal-oriented rehabilitation [19] and early-stage dyadic interventions [20] support self-management skills by helping people to manage the present and future impact of the condition, identify and implement memory management strategies and plan ahead to take control of legal, financial and health issues. Therefore, there are preliminary, but limited, indications that people with early-stage dementia could benefit from a more focused self-management approach.

While self-management approaches have common components across conditions, these approaches are applied with subtly different emphases depending on the nature of the condition in question; for example, in diabetes there is a focus on self-medicating and self-monitoring [21] while the focus is on managing symptoms and promoting behaviour change in chronic obstructive pulmonary disease [22], asthma [23] and rheumatism [24]. In order to conceptualise self-management in early-stage dementia, it will be important to adapt the approach to take account of condition-specific factors. However, there is limited evidence on which to base the development of self-management approaches for people with dementia. One study explored what people with dementia think should be included in self-management interventions. Although few details were provided about the participants who contributed, suggested emphases included managing dementia alongside other conditions, managing unexpected symptoms, and the importance of maintaining meaningful roles [25]. Two further papers have examined views about self-management held by a range of respondents, including both health professionals and people with dementia. Perceived barriers to self-management included the impact of societal views and public impressions and a general lack of information [26]. Concepts of self-management echoed elements of person-centred care and emphasised the ‘self’ element of self-management by focusing on managing life with dementia rather than managing the dementia itself [27]. However, in these two papers the views of people with dementia were not reported separately from the insights gained from other respondents [26,27]. There is a need for further in-depth investigation of the views of people with dementia and caregivers to help formulate a dementia-specific approach to self-management, which can then be evaluated in terms of feasibility, acceptability and clinical efficacy, and ease of use by clinical teams. The SMART study aims to address this need.

In the first phase of the SMART study, following an initial qualitative investigation in which the perspectives of people with dementia and caregivers were examined, and a review of the literature on the theory and practice of self-management in a range of conditions, a manual for an eight-session group self-management intervention for people with early-stage dementia was developed. The intervention approach is based on self-regulation models and social cognitive theory, and the overall aim is to enhance participants’ self-efficacy and problem-solving skills through focusing on meaningful and relevant aspects of living with dementia. The pilot trial outlined in this protocol is intended to provide preliminary evidence about the feasibility and efficacy of the intervention so that a subsequent full-scale randomised controlled trial (RCT) may be powered from the best evidence available in order to address the following objectives.

The primary objective is to evaluate the effectiveness of the self-management intervention in improving self-efficacy in people with dementia compared to treatment as usual (TAU). The secondary objectives are to evaluate the effectiveness of the self-management intervention in (a) improving mood, well-being and cognitive test scores in people with dementia compared to TAU; (b) decreasing caregiver stress and general distress compared to TAU, and (c) estimating the cost-effectiveness of the self-management intervention compared to TAU and establishing whether it results in a reduction in health service utilisation.

## Methods

### Design

**Figure 1:**
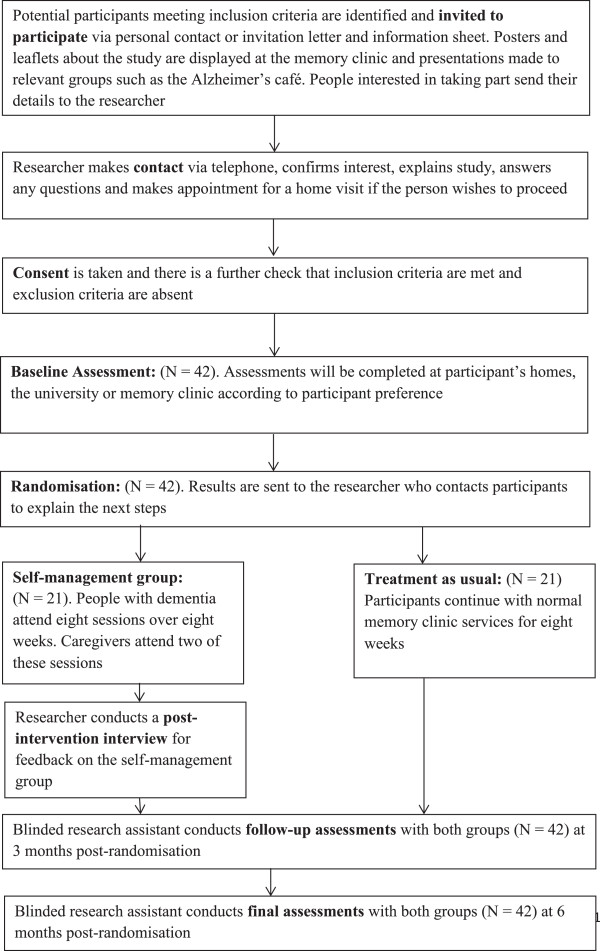
Consort diagram.

This SMART study involves two phases, following the development and feasibility/piloting phases of the Medical Research Council guidelines for complex interventions [28] and the present paper relates to phase II. In phase I of the study we triangulated evidence from a systematic review and qualitative analysis of interviews with people with dementia and caregivers to inform the development of a protocol for a self-management group intervention. In phase II we will evaluate the group intervention in a single-site, single-blind pilot RCT comparing the self-management group with TAU. Outcomes will be assessed at 3 and 6 months post-randomisation by a researcher blinded to group allocation (Figure 1, CONSORT diagram).

### Setting and participants

The aim is to recruit 42 people with early-stage dementia and their primary caregivers. The study will proceed in three waves with approximately 14 participants in each wave. The sample size will provide adequate statistical power for a pilot evaluation of the self-management group, and decisions about sample size have been informed by the need to balance the number of participants required to provide an appropriate amount of information for a pilot study with the number that it is feasible to recruit within the study timescale. As this is a pilot trial, particular note will be taken of recruitment and retention rates so that a future definitive study may be accurately powered. All participants will need to provide informed consent. In order to be deemed to provide informed consent, participants will have to demonstrate that they can understand the study information, retain and weigh up this information sufficiently to clearly communicate their decision about participation, and clearly understand the consequences of this decision.

Participants will be identified from a memory clinic in a semi-rural area of North Wales. The inclusion criteria for people with dementia are as follows: 1) participants must be diagnosed with Alzheimer’s, vascular or mixed Alzheimer’s and vascular dementia, according to ICD-10 criteria [29] and in the early stages as indicated by a score of 20 or more on the Mini-Mental State Examination [30]. These diagnoses account for 89% of dementia diagnoses [31] and rarer sub-types of dementia have been excluded here because they involve features that would require a specific approach; 2) participants must be able to provide informed consent; 3) participants may be either taking or not taking acetyl-cholinesterase inhibitors or Memantine. Participants receiving acetyl-cholinesterase inhibitors or Memantine must have been stabilised on their current dose for a minimum of one month prior to baseline assessment, with no plan to change dosage or medication during the course of the study. This is to ensure that change is not confounded by medication effects, and 4) participants must have a caregiver who is willing to participate in the study. Caregivers may be spouses, partners, siblings or adult children of people with early-stage dementia who are involved in providing day-to-day support.

The exclusion criteria for people with dementia are as follows: 1) a history of stroke, significant neurological or psychiatric conditions (for example, psychosis) or brain injury. These conditions may affect cognitive, emotional and behavioural functioning and thus act as confounders; 2) current significant anxiety or depressive disorder that would affect cognitive, emotional and behavioural functioning and thus act as confounders, and 3) inability to speak English sufficiently well to allow completion of the assessment measures.

In addition, participants who are currently attending other group-based psychosocial interventions will not be enrolled in the study until these have been completed.

There are no specific exclusion criteria for caregivers; any caregiver willing to take part together with the person with dementia for whom she or he provides care will be eligible for inclusion.

### Outcome measures

#### Primary outcome measure

The primary outcome measure will be the scores recorded by participants with dementia on the General Self-Efficacy Scale (GSES) [32] at 3 months post-randomisation. The GSES aims to measure a person’s broad and stable sense of personal competence to deal effectively with a variety of stressful situations. The current version of the scale contains 10 items and participants can complete the measure in approximately 4 minutes. The GSES has high reliability, stability, construct and factorial validity. It has demonstrated convergent and discriminant validity [33].

#### Secondary outcome measures completed by people with dementia at baseline, and three and six months post randomisation

An important secondary outcome is the GSES score at 6 months post-randomisation, which will allow us to evaluate whether the effects of the self-management group extend into the medium term.

##### Cognitive functioning

The Addenbrooke’s Cognitive Examination - III (ACE-III) [34] is a brief test sensitive to the early stages of dementia. It measures cognitive ability in five domains: attention (four items), memory (five items), fluency (two items), language (eight items) and visuo-spatial ability (five items). It is administered by the researcher and can be completed in approximately 12–20 minutes. The ACE III has demonstrated convergent validity and internal reliability. It has high sensitivity and specificity in detecting cognitive problems [35].

##### Anxiety and depression

The Hospital Anxiety and Depression Scale (HADS) [36,37] is a self-report measure of symptoms of anxiety and depression. All symptoms that can also relate to physical disorder, such as fatigue, have been excluded, which makes the scale particularly suited to older adults who may experience other medical conditions. It contains 14 items each answered on a 4-point Likert scale (0 = not at all, 3 = very often indeed) though some items are reverse-scored. Subtotals are derived for anxiety and depression. The HADS has been previously employed and validated in people with dementia [38].

##### Well-being/risk

The Clinical Outcomes in Routine Evaluation - Outcome Measure (CORE-OM) [39] is a self-report measure of problem severity. It contains 34 items which are scored on a 4-item Likert scale (0 = not at all, 4 = most or all of the time). It covers four domains: wellbeing, social functioning, problems/symptoms, and risk to self and risk to others, as well as providing a global distress score. The CORE-OM has good internal consistency and test-retest reliability. It also has good convergent validity and shows sensitivity to change [39].

##### Health-related quality of life

The EQ-5D-3L [40,41] was designed to measure health-related quality of life. The assessment has five questions covering five domains (mobility, self-care, usual activities, pain/discomfort and anxiety/depression). Each item is answered on a 3-point scale (level 1 = no problems, level 3 = unable to do or extreme problem doing). A visual analogue scale is also incorporated into the measure and respondents indicate how good their health is at the time of rating on a 0-to-100 scale, with the anchor points being worst (0) and best (100) imaginable health state. The EQ-5D-3L has been used in a UK sample of people with dementia, including early-stage dementia [42]. A systematic review of recent studies using the EQ-5D-3L with people with dementia reported good feasibility and reliability of the instrument [43]. In a French sample of people with dementia the measure has demonstrated acceptability and construct validity, although inter-rater agreement is limited [44]. In other populations the EQ-5D-3L has good test-retest reliability [41].

##### Quality of life

The instrument, the ICEpop CAPability Measure for Older People (ICECAP-O) [45] focuses on a broader sense of wellbeing. It covers the attributes of quality of life that were rated as important by a UK sample of older people. It measures five attributes (attachment, role, enjoyment, security and control) on a 4-point scale (4 = I can have/I am able, 1 = I cannot have/I am not able). This scale has demonstrated acceptability in a sample of UK older adults and there is initial evidence of construct validity [46].

##### Service use

The Client Socio-Demographic and Service Receipt Inventory (CSRI) [47] asks participants about their use of health and social services over the last three months. They are asked to report the frequency and intensity of their service use. It takes approximately 20 minutes to complete. The CSRI has adequate concurrent validity [47]. In the current study, if necessary, the caregiver will be asked to help the person with dementia to complete this measure.

#### Secondary outcome measures completed by caregivers at baseline, and three and six months post-randomisation

##### Caregiver distress

The Neuropsychiatric Inventory Questionnaire (NPI-Q) [48] assesses behavioural and psychological symptoms and covers 12 domains. The 12 items are scored for severity (3-point scale) and for the degree of caregiver distress experienced (5-point scale). It is a self-report measure and can be completed in approximately 5 minutes. The NPI-Q has good internal consistency and test-retest reliability. It has demonstrated construct validity [48].

##### Caregiver stress

The Relative Stress Scale (RSS) [49] is a self-report measure of caregivers’ levels of stress relating to caring for their relative. There are 15 items answered on a 5-point Likert scale (0 = not at all, 4 = always/considerably). The scale comprises three subscales: personal distress, life upset from caregiving and negative feelings. The RSS has factorial validity and adequate internal consistency [50].

##### Health-related quality of life

The EQ-5D-3L [40,41] will be used (see above).

##### Quality of life

The ICECAP-O [45] will be used (see above).

##### Service use

The CSRI [47] will be used (see above).

##### Post-intervention interview

Participant dyads randomised to the self-management group will be invited to take part in a 20-minute semi-structured interview to explore their perceptions and views of the group. This interview will be conducted by the researcher and will be audio-recorded.

### Intervention

The self-management group intervention will involve eight 90-minute sessions held at weekly intervals at a memory clinic in North Wales. It will be led by two members of the clinical team who will be trained to administer the intervention by the research team, with ongoing access to support during the study. Up to seven people with dementia will attend each group, and caregivers (a relative or friend of each participating person with dementia) will be invited to attend the first and final sessions. Caregivers may also, if they wish, join the group at the end of each meeting to hear an overview of the theme that has been covered. Participants will be invited to share only personal information that they are comfortable disclosing during the self-management group. Each attendee will receive a booklet which will cover the content of each session: this will allow space for additional notes and comments to be made and the person with dementia can share this resource with the caregiver between sessions. Participants attending the self-management group will continue with all other care services they are receiving.

There will be a flexible approach to the organisation and structure of sessions. Each session will cover a particular theme and participants will discuss the theme with each other and the facilitators. Within each theme participants will be able to focus on aspects that are meaningful to group members. Session themes will cover an orientation to the group and information sharing, enjoying hobbies, activities and interests, staying well, practical ways to manage memory difficulties, maintaining relationships and social networks, planning for the future, coping skills and accessing local resources. The group will be facilitated in an informal manner and time will be provided for more social activities. The facilitators will keep a record of the number of intervention sessions attended by each participant. The facilitators will also write a summary of each session incorporating their impressions of participants’ involvement in the meeting.

### Comparison condition: treatment as usual

Participants randomised to TAU will continue to receive the normal services provided by the memory clinic. These services include a regular nurse-led clinical review and access to services such as psychiatry, occupational therapy and social services as needed. Using TAU as a comparator condition ensures that all participants receive needed services.

### Procedure

Potential participants will be invited to take part via an invitation letter or via personal contact from a member of the clinical team. Posters and leaflets advertising the study will be displayed at the memory clinic and groups that make use of the service facilities, such as the local Alzheimer’s café, will receive information about the study. As this is a pilot study, a range of recruitment methods is envisaged to inform future multi-centre trials. People interested in taking part will be contacted by a researcher who can provide more information about the study and answer any questions. If the person is interested in taking part in the study the researcher will arrange a meeting. If the person consents to take part in the study, the researcher will make final eligibility checks and carry out the baseline assessments. Participants will be allowed to pace these assessment visits according to their needs. For instance, short breaks will be permissible if participants are fatigued and if necessary two visits will be made to complete the assessments at each data point.

Eligible, consenting participants will be randomised to receive either the self-management group intervention or TAU after baseline assessments. Randomisation will be conducted by the North Wales Organisation for Randomised Trials in Health (NWORTH). Randomisation will be undertaken using a computer-based algorithm. Randomisation will be balanced (one: one dynamic allocation) [51] and will be stratified for mini-mental state examination score (20 to 24, 25 to 25+) and gender.

Follow-up assessments will occur at 3 and 6 months post-randomisation and will be undertaken by a researcher blinded to group allocation. There will be no unblinding of the researcher, and participants will be asked not to tell the researcher whether they attended the group. To assist retention in the study, appointments will be prescheduled with participants, and reminders of upcoming meetings will be sent. Participants who attend the self-management group will additionally be invited to take part in a post-intervention interview to feed back their experiences of attending the group.

### Analysis

Data will be entered into IBM SPSS Statistics v20 and checked to ensure that accuracy is within acceptable limits. Participants who have completed assessments for at least two data points will be included in the analysis. All available data will be included and an intention-to-treat analysis will be conducted. If an outcome measure has less than 20% missing responses, missing data will pro-rated with the participant’s mean item score to allow a calculation of the total score. If an outcome measure has more than 20% missing responses, methods of multiple imputation will be explored and a range of sensitivity analyses will be performed to inform the main RCT. As this is a pilot study all outcomes will be reported and particular note will be taken of effect sizes and confidence intervals.

Analysis of covariance (ANCOVA) using baseline scores and stratification variables as covariates will compare group outcomes with regard to the primary outcome, the self-efficacy scores of people with dementia at 3 months post-randomisation. Secondary outcomes will be analysed similarly and confidence intervals will be quoted for all parameter estimates. The Bonferroni correction for multiple testing will not be made as this is a pilot exploratory study to estimate potential effect sizes rather than a strictly hypothesis-testing experimental design. The post-intervention interviews will be analysed using thematic analysis to explore and summarise participants’ experience of the intervention. This analysis will be undertaken independently by members of the research team. Inter-rater reliability will be addressed by having a random selection of 20% of transcripts coded by two raters so that differences can be discussed and resolved. If inter-rater reliability reaches 80% or more, then the remainder of transcripts can be coded by a single rater. If not, a further 20% of transcripts will be coded by both raters and the process repeated.

An economic evaluation will investigate the incremental cost-effectiveness of the self-management group compared with TAU. Costs for the group will be calculated from a public-sector multi-agency perspective. Primary and secondary care health service use will be collected using the CSRI and costs will be calculated using national unit costs [52,53]. A cost-utility analysis using the EQ-5D-3L will generate a cost per quality-adjusted life-year and cost-effectiveness acceptability curve for comparison with the National Institute for Health and Care Excellence ceiling of £20,000 to £30,000 [54]. A secondary exploratory cost-effectiveness analysis will be undertaken using the GSES and ICECAP-O as the measures of effectiveness. As this is a pilot trial with a relatively small sample size, the tree-age modelling package will be utilised to map a decision analytic model to inform data collection for the economic evaluation of a future definitive multi-centre study.

### Ethical approval

The study has ethical approval from the North Wales Research Ethics Committee-West (Reference: 13/WA/0174) and the School of Psychology Ethics Committee, Bangor University. All data, including interview transcripts, will be anonymised. Participants will also be made aware that they can omit any questions they do not wish to answer during the assessment sessions and will be made aware of their right to withdraw from the study at any point. Participant information will be stored securely at the university site and all electronic data will be encrypted. Anonymised data will be kept for up to 5 years after the end of the study. Only the research team will have access to the full dataset.

## Discussion

At present the evidence base for self-management in people with early-stage dementia is minimal. Given the potential of people with early-stage dementia to develop self-management skills, it is timely to consider how health services may support this process. This study will implement a self-management group intervention and evaluate its efficacy and cost-effectiveness in a pilot randomised controlled trial, conducted within a National Health Service memory clinic in the UK. This will provide preliminary evidence about the usefulness and acceptability of such an approach to people with early-stage dementia and their caregivers, which will serve as a basis for further research.

## Trial status

The trial is ongoing and is due to finish in December 2014.

## Abbreviations

ACE-III: Addenbrooke’s Cognitive Examination-III; CORE-OM: Clinical Outcomes in Routine Evaluation-Outcome Measure; CSRI: Client Socio-Demographic and Service Receipt Inventory; EQ: Euroqol; GSES: General Self-Efficacy Scale; HADS: Hospital Anxiety and Depression Scale; ICECAP-O: The ICEpop CAPability Measure for Older People; NPI-Q: Neuropsychiatric Inventory Questionnaire; NWORTH: North Wales Organisation for Randomised Trials in Health; RCT: randomised controlled trial; RSS: Relative Stress Scale; TAU: treatment as usual.

## Competing interests

The authors declare that they have no competing interests.

## Authors’ contributions

CQ: study design, preparation and drafting of study protocol, drafting of manuscript. DA: study design, preparation of study protocol, and review of manuscript. GT: trial management, recruitment and assessment of participants, drafting of manuscript. RhW: study design, preparation of study protocol, statistical analysis plan, review of manuscript. RTE: study design, preparation of study protocol, health economics analysis plan, review of manuscript. CJ: revision of health economics analysis plan, review of manuscript. LC: study concept, study design, preparation and drafting of study protocol, drafting of manuscript. All authors contributed to the refinement of the study protocol and all authors have read and approved the final manuscript.

## Authors’ information

CQ (BSc, MSc, PhD) works as a Research Fellow at the School of Psychology, Bangor University, Bangor, Gwynedd, LL57 2AS. DA (MB, ChB, MA, MRCPsych) was a Consultant Old Age Psychiatrist at Glan Traeth Day Hospital. He is now Medical Director and Consultant Psychiatrist at The Retreat, Heslington Road, York, YO10 5BN. GT (D.Clin.Psy, PGCE, BSc) works as a Research Officer at the School of Psychology, Bangor University, Bangor, Gwynedd, LL57 2AS. RhW (BSc, MSc, Cstat, Csci) is associate director at the North Wales Organisation for Randomised Trials in Health, Y Wern, Bangor University, The Normal Site, Holyhead Road, Gwynedd, UK, LL57 2PZ. RTE (BSc, MSc, PhD) is co-director at the Centre for Health Economics and Medicines Evaluation, Bangor University, Dean Street, Gwynedd, LL57 1UT. CJ (BSc, PhD) is a Research Officer at the Centre for Health Economics and Medicines Evaluation, Bangor University, Dean Street, Gwynedd, LL57 1UT. LC (MA, MSc, PhD, CPsychol) is Professor of Clinical Psychology and Neuropsychology at the School of Psychology, Bangor University, Bangor, Gwynedd, LL57 2AS.
